# Supplementary material for: A set of multi-entry identification keys to African frugivorous flies (Diptera, Tephritidae)
Source: Zookeys. 2014 Jul 24;(428):97–108. doi: 10.3897/zookeys.428.7366 (PMC4143993; doi:10.3897/zookeys.428.7366)
Supplement: Supplementary material 6 — Key to Ceratitis [file zookeys-428-097-s006.zip › SF6_ZooKeys_key to Ceratitis/key/SF6_ZooKeys_key to Ceratitis/Media/Html/Ceratitis venusta .htm]

Ceratitis (Ceratalaspis) venusta (Munro)


***Ceratitis*** **(*Ceratalaspis*)
*venusta* (Munro)**

*Hoplolophomyia venusta* Munro, 1956: 466.

Body
length: 5.60 (5.10-5.85) mm; wing length: 5.77 (5.40-5.90) mm.

Male

Head.
Antenna yellow. Third antennal segment twice as long as second segment. Arista
with short hairs over entire length. Frons with short scattered hairs which are
largely the same colour as frons; convex, in lateral view not distinctly
projecting forwards at antennal implant. Chaetotaxy normal for subgenus.

Thorax.
Postpronotum white, unspotted. Ground colour of mesonotum pale with orange
tinge; mesonotal pattern, median spot between postpronota usually absent,
sometimes present as darker patch. Chaetotaxy normal for subgenus. Scapular
setae black. One anepisternal bristle. Scutellum white basally, otherwise
yellow with three black separate markings apically, hardly visible in dorsal
view, restricted to apical margin; basally without dark spots, only slight
darker colouration. Subscutellum completely dark.

Legs
yellow; setation typical for subgenus, mainly pale especially on femora.
Posterior and posterodorsal rows on fore femur pale.

Wing
bands markings extensively yellow. Banding, marginal band with clear
interruption after cell R1; cubital and medial bands joined with marginal band
(exceptionaly the cubital band is separate); crossvein r-m at middle of discal
cell. Crossvein dm-cu posteriorly more inwards than anteriorly.

Abdomen.
Yellow. Setation and banding typical for subgenus.

Female

As
male except for the following characters: Ocellar bristles sometimes shorter,
at most 1.5 times length of ocellar triangle. Occasionally only one frontal
bristle. Lower eye margin sometimes with darker colouration. Posterior and
posterodorsal rows on fore femur apically with dark hairs. Crossvein r-m before
middle of discal cell. Oviscape shorter than abdominal terga 3-6 combined (in
one specimen longer than terga). Aculeus with tip variable.

(Description
after De Meyer, 1998)
